# The pathogenesis of low pathogenicity H7 avian influenza viruses in chickens, ducks and turkeys

**DOI:** 10.1186/1743-422X-7-331

**Published:** 2010-11-19

**Authors:** Erica Spackman, Jack Gelb, Lauren A Preskenis, Brian S Ladman, Conrad R Pope, Mary J Pantin-Jackwood, Enid T Mckinley

**Affiliations:** 1 Southeast Poultry Research Laboratory, USDA-ARS, 934 College Station Rd. Athens, GA, 30605, USA; 2 Avian Biosciences Center, University of Delaware, Newark, DE, 19716, USA

## Abstract

**Background:**

Avian influenza (AI) viruses infect numerous avian species, and low pathogenicity (LP) AI viruses of the H7 subtype are typically reported to produce mild or subclinical infections in both wild aquatic birds and domestic poultry. However relatively little work has been done to compare LPAI viruses from different avian species for their ability to cause disease in domestic poultry under the same conditions. In this study twelve H7 LPAI virus isolates from North America were each evaluated for their comparative pathogenesis in chickens, ducks, and turkeys.

**Results:**

All 12 isolates were able to infect all three species at a dose of 10^6 ^50% egg infectious doses based on seroconversion, although not all animals seroconverted with each isolate-species combination. The severity of disease varied among isolate and species combinations, but there was a consistent trend for clinical disease to be most severe in turkeys where all 12 isolates induced disease, and mortality was observed in turkeys exposed to 9 of the 12 viruses. Turkeys also shed virus by the oral and cloacal routes at significantly higher titers than either ducks or chickens at numerous time points. Only 3 isolates induced observable clinical disease in ducks and only 6 isolates induced disease in chickens, which was generally very mild and did not result in mortality. Full genome sequence was completed for all 12 isolates and some isolates did have features consistent with adaptation to poultry (e.g. NA stalk deletions), however none of these features correlated with disease severity.

**Conclusions:**

The data suggests that turkeys may be more susceptible to clinical disease from the H7 LPAI viruses included in this study than either chickens or ducks. However the severity of disease and degree of virus shed was not clearly correlated with any isolate or group of isolates, but relied on specific species and isolate combinations.

## Background

Avian influenza (AI) virus causes one of the most economically important diseases of poultry worldwide. AI is classified by the world organization for animal health (OIE) into two forms, low pathogenicity (LP) and high pathogenicity (HP), based on virulence in chickens [1]. H7 is one of the two most economically important AI virus subtypes because historically all HP AI viruses have been either the H7 or H5 subtype and it is among the most common subtypes in commercial poultry in the world [1,2]. In numerous cases the HP form mutated from a LPAI H7 (or H5) virus that was circulating in chickens or turkeys [3-6]. However, not all H7 LPAI viruses become HP.

In the U.S., H7 AI viruses are sporadically recovered from wild birds (WB) and commercial poultry. Many of the outbreaks in commercial poultry [7-9] can be traced to the live bird market (LBM) system of New York and New Jersey where a single LP H7 genetic lineage persisted from 1994 to 2006 [10,11]. Few studies have directly compared the pathogenesis of AI virus in the three primary poultry species: chickens, ducks and turkeys. The aim of this work was to characterize the pathogenesis of selected North American H7 LPAI virus isolates from WB, commercial poultry, and the LBMs in the three primary domestic poultry species; chickens, ducks and turkeys.

## Results

### Clinical disease

**Figure 1:**
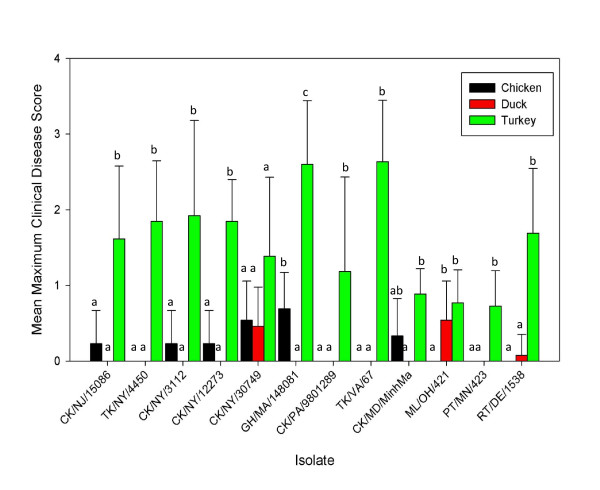
**Mean maximum clinical disease score for chickens, ducks and turkeys infected with H7 LPAI virus**. Clinical signs were scored as: 0 = no signs, 1 = mild to moderate respiratory signs (mild depression in ducks), 2 = moderate to severe (i.e. depressed, not eating, neurological signs), 3 = Dead. Letters denote statistical groups among species for each virus. Error bars indicate standard deviation of clinical disease scores.

Clinical disease signs varied in severity among the virus-host combinations. Mean maximum clinical disease scores (the mean of the maximum clinical scores for each bird) ranged from 0 to 0.7 in chickens (Figure 1). Disease was only observed in chickens with 6 isolates (Figure 1) and not all chickens in these groups were affected. Disease signs in chickens were primarily conjunctivitis and lacrimation, which generally occurred from 2-4 days post infection. Only 3 isolates caused observable clinical disease in ducks (CK/NY/30749, ML/OH/421 and RT/DE/1538 (abbreviations defined in table 1)). Mean maximum clinical disease scores for ducks ranged from 0 to 0.5. The primary clinical sign presented by ducks was nasal discharge at 2 days PI and conjunctivitis. All 12 isolates caused observable clinical disease in turkeys with mean maximum scores ranging from 0.7 to 2.6, which were significantly higher than chickens and ducks with 8 of the 12 viruses. Clinical disease in turkeys included mild to severe conjunctivitis, nasal discharge, swollen sinuses as well as lethargy. Turkeys were the only species where mortality was observed, which ranged from 10-60% with 9 isolates (Table 2). The turkeys that died had severe sinusitis (a bacteriological examination was not conducted). Only one isolate, CK/NY/30749, caused clinical disease in all three species, although shed titers and clinical disease was most severe in turkeys. At no time were clinical disease signs observed in any of the sham inoculated birds.

**Table 1:** Low pathogenicity avian influenza virus isolates evaluated for pathogenesis in chickens, ducks and turkeys.

Isolate	Subtype	Source	HA cleavage site	NA stalk deletion	NS subtype	Abbreviation
A/chicken/NJ/15086-3/1994	H7N3	LBM^A^	PENPKT/R	None	A	CK/NJ/15086
A/turkey/NY/4450-4/1994	H7N2	LBM	PENPKT/R	50-73^B^	B	TK/NY/4450
A/chicken/NY/3112-1/1995	H7N2	LBM	PENPKP/R	58-73	B	CK/NY/3112
A/chicken/NY/12273-11/1999	H7N3	LBM	PENPKT/R	None	A	CK/NY/12273
A/chicken/NY/30749-3/2000	H7N2	LBM	PEKPKP/R	None	B	CK/NY/30749
A/guinea hen/MA/148081-11/2002	H7N2	LBM	PEKPKK/R	58-73	B	GH/MA/148081
A/chicken/PA/9801289/1998	H7N2	Commercial-poultry (LBM lineage)	PENPKP/R	58-73	B	CK/PA/9801289
A/turkey/VA/SEP-67/2002	H7N2	Commercial-poultry (LBM lineage)	PEKPKP/R	58-73	B	TK/VA/67
A/chicken/MD/MinhMa/2004	H7N2	Commercial-poultry (LBM lineage)	PEKPKP/R	58-73	B	CK/MD/MinhMa
A/mallard/OH/421/1987	H7N8	WB	PESPKT/R	None	A	ML/OH/421
A/pintail/MN/423/1999	H7N3	WB	PENPKT/R	None	A	PT/MN/423
A/ruddy turnstone/DE/1538/2000	H7N9	WB	PENPKT/R	None	A	RT/DE/1538

A. LBM = live bird market; WB = wild bird B. Range of residues deleted.

**Table 2:** Mortality and mean death time in turkeys inoculated with North American H7 LPAI virus Isolates.

Isolate	Mortality	Mean death time (days)
CK/NJ/15086	40 (4/10)^A^	7.0
TK/NY/4450	10 (1/10)	12.5
CK/NY/3112	60 (6/10)	9.1
CK/NY/12273	10 (1/10)	13.0
CK/NY/30749	20 (2/10)	9.0
GH/MA/148081	50 (4/8)	7.75
CK/PA/9801289	0 (0/8)	NA^B^
TK/VA/67	33.3 (3/9)	10.0
CK/MD/MinhMa	12.5 (1/8)	5.0
ML/OH/421	0 (0/10)	NA
PT/MN/423	0 (0/8)	NA
RT/DE/1538	10 (1/10)	5.0

A. Percent (Number dead/total)

B. NA = not applicable

Microscopic lesions were consistent with what has been previously reported for AI virus in chickens, ducks and turkeys. Briefly, lesions observed in tissues from turkeys included viral tracheitis, cilial loss, heterophilic cecitis, and heterophilic rhinitis in the nasal cavity. Patchy cilial loss in the trachea, heterophilic tracheitis, and serositis of the kidney was observed in tissues from ducks. Rare to minimal rhinitis and the presence of heterophils, excessive mucus and slight lymphocytic accumulation were observed in the nasal cavity as well as rare focal heterophilic bronchitis was seen in tissues from chickens.

### Virus shed

**Figure 2:**
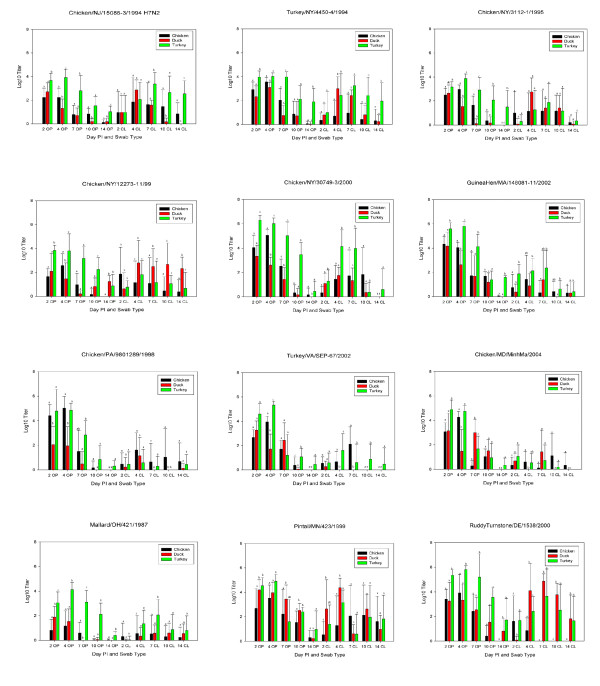
**Mean OP and CL virus shed titers from white leghorn chickens, Pekin ducks and broad-breasted white chickens by day PI as determined by quantitative real-time RT-PCR for the influenza M gene**. Letters indicate statistical groupings among the 3 species for each day PI and swab type. Error bars indicate standard deviation of titers. Abbreviations CL = cloacal, OP = oropharyngeal.

At days 2 PI, oro-pharyngeal (OP) and cloacal (CL) swabs from all species were positive for AI viruses by quantitative real-time RT-PCR (qrRT-PCR) with the exception of CL swabs from ducks and turkeys inoculated with ML/OH/421 (Figure 2). However, by day 4 PI CL swabs from ML/OH/421 infected ducks and turkeys were positive. The highest OP titers were observed 2-4 days PI, although in ducks, viral shed tended to peak at 2 days PI, whereas in chickens and turkeys, shed tended to peak at 4 days PI. The highest mean OP titer for turkeys corresponds to a titer of 10^6.3 ^50% egg infectious doses (EID_50_) with CK/NY/30749 at 2 days PI and GH/MA/148081 at 4 days PI (Figure 2). The highest mean OP titer from chickens was 10^5.8^EID_50 _at 4 days PI with CK/PA/9801289 (Figure 2) and the highest mean duck OP titer was 10^4.9^EID_50 _at 2 days PI with PT/MN/423. Titers from CL swabs were consistently lower than OP titers and were generally higher from ducks and turkeys than from chickens with peak titers observed between 4-7 days PI. The highest CL shed titers were 10^3.8^EID_50 _with RT/DE/1538 for turkeys at 7 days PI, 10^2.9^EID_50 _with TK/VA/67 at 7 days PI in chickens, and 10^5.1^EID_50 _with PT/MN/423 at 4 days PI from ducks. Oral and CL shed persisted 'through 10 and 14 days PI with most isolate and species combinations (Figure 2).

When comparing species for shed of all viruses collectively, turkeys shed significantly (p < 0.05) more virus orally than either chickens or ducks at 2, 7 and 14 days PI. At 4 days PI the amount of virus shed orally by both chickens and turkeys was significantly higher than ducks. Ten days PI OP viral shed by turkeys was significantly higher than chickens. The only statistically significant differences among species in CL shed for all viruses collectively was at 14 days PI when turkeys shed higher titers than either chickens or ducks. All sham inoculated birds were negative for AI virus OP and CL shedding throughout the experiment.

### Immunohistochemistry

**Figure 3:**
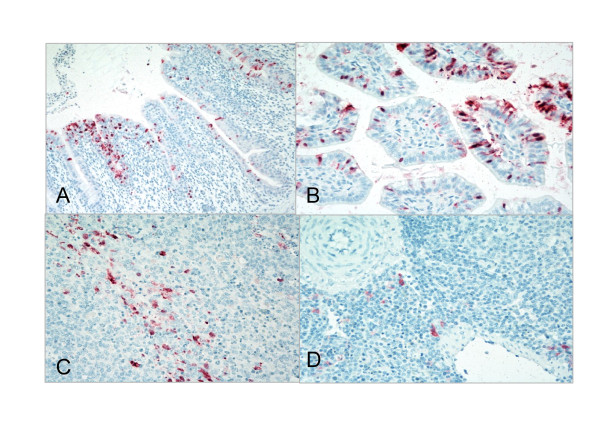
**Immunohistochemical staining for avian influenza virus antigen in tissues of chickens, turkeys and ducks infected with H7 AI viruses, 2 days PI**. A. Viral antigen (red staining) in bronchial epithelial cells from a turkey infected with TK/VA/67. B. and C. Viral antigen (red staining) in the intestinal epithelium and cecal tonsils of a chicken infected with CK/NY/3112. D. Viral antigen (red staining) in macrophages in the spleen of ducks infected with TK/VA/67.

In order to evaluate sites of virus replication, immunohistochemical (IHC) staining for AI virus antigen with an antibody to the influenza A NP protein was conducted with tissues collected 3 days PI selected from birds shedding more than 10^4^EID_50 _at 2 days PI (Figure 3). The bronchial epithelium of turkeys inoculated with CK/MD/MinhMa, CK/NJ/15086 or TK/VA/67, and the airsac epithelium of a turkey exposed to TK/NY/4450 were positive for AI virus staining. Avian influenza virus antigen was observed in macrophage in spleens from ducks exposed to GH/MA/148081 or TK/VA/67, and both the bronchus and spleen from ducks infected with PT/MN/423 were positive. Staining of macrophage in the spleens of AIV infected ducks has been reported before [12]. However it is not clear whether the virus is replicating or whether the antigen is present from non-specific uptake of the virus. The intestines and cecal tonsils of chickens infected with CK/NJ/15086 or CK/NY/3112 were also positive for AI virus antigen by immunohistochemical staining.

### Serology

At the termination of the experiments, 18-21 days PI, blood was collected from surviving birds and AI virus antibody was evaluated by commercial ELISA for all 3 species. All surviving turkeys except one exposed to CK/MD/MinhMa had detectable AI virus antibody at termination (Table 3). All of the chickens seroconverted to all isolates except PT/MN/423 (89%), CK/PA/9801289 (90%), ML/OH/421 (70%) and RT/DE/1538 (90%). The proportion of ducks which serocoverted varied by isolate, and 100% of exposed ducks seroconverted to only 5 isolates, seroconversion to the remaining isolates were between 20% (CK/NY/30749) and 90% (TK/NY/4450) (Table 3).

**Table 3:** Avian influenza virus antibody in sera from chickens, ducks and turkeys by commercial ELISA at termination of the experiment.

Species	Isolate	ELISA	
		
		Percent	Pos/total
Chicken	CK/NJ/15086	100	10/10
	TK/NY/4450	100	10/10
	CK/NY/3112	100	10/10
	CK/NY/12273	100	10/10
	CK/NY/30749	100	9/9
	GH/MA/148081	100	10/10
	CK/PA/9801289	90	9/10
	TK/VA/67	100	8/8
	CK/MD/MinhMa	100	10/10
	ML/OH/421	70	7/10
	PT/MN/423	89	8/9
	RT/DE/1538	90	9/10
			
Duck	CK/NJ/15086	100	10/10
	TK/NY/4450	90	9/10
	CK/NY/3112	70	7/10
	CK/NY/12273	100	10/10
	CK/NY/30749	20	2/10
	GH/MA/148081	50	5/10
	CK/PA/9801289	44	4/9
	TK/VA/67	63	5/8
	CK/MD/MinhMa	33	2/6
	ML/OH/421	100	10/10
	PT/MN/423	100	9/9
	RT/DE/1538	100	10/10
			
Turkey	CK/NJ/15086	100	10/10
	TK/NY/4450	100	8/8
	CK/NY/3112	100	4/4
	CK/NY/12273	100	9/9
	CK/NY/30749	100	8/8
	GH/MA/148081	100	4/4
	CK/PA/9801289	100	8/8
	TK/VA/67	100	6/6
	CK/MD/MinhMa	86	6/7
	ML/OH/421	100	10/10
	PT/MN/423	100	6/6
	RT/DE/1538	100	9/9

### Sequencing and phylogenetic analysis

**Figure 4:**
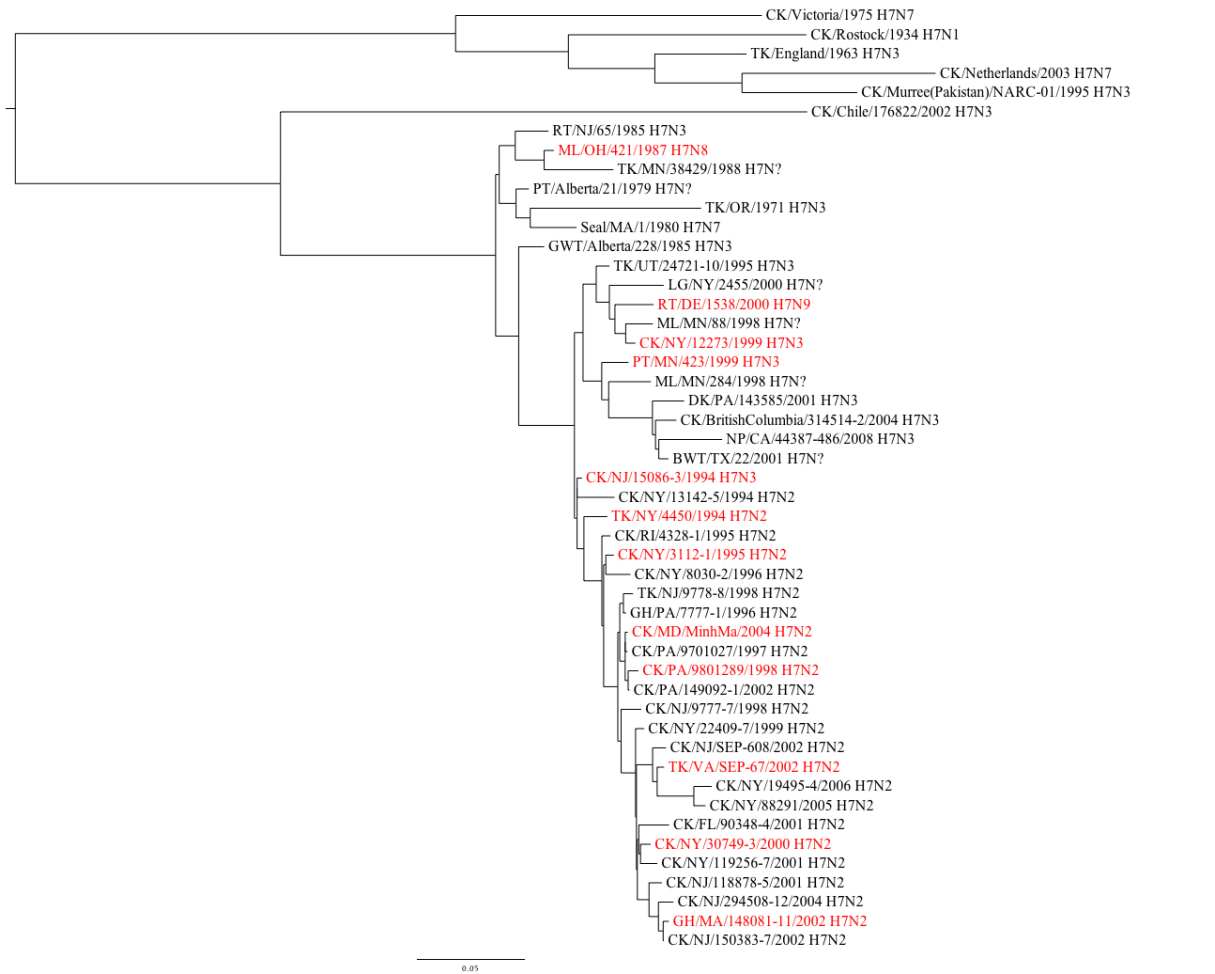
**Phylogenetic tree of the H7 HA gene of influenza virus**. Isolates included in this study appear in red type.

Full genome sequence was generated for all 12 isolates to evaluate their genetic diversity and origins. Genetic diversity of the HA gene of domestic H7 isolates was one criteria used to select the isolates for this study, therefore the sequences of the HA genes of many of the isolates used here have been previously reported [10,11]. Based on these reports and the known epidemiology of the viruses, the H7 genes had been classified into 2 groups: 1). LBM and LBM-poultry, and 2). WB, which contained one isolate from the LBM system CK/NY/12273 (Figure 4). The isolates in this study have a range of nucleotide (nt) identity of 91.1 to 98.9% among their HA genes. Three proteolytic cleavage sites (PCS) were observed among the 12 isolates, all of which are consistent with LPAIV (Table 1). Five isolates (CK/NY/30749, GH/MA/148081, CK/MD/MinhMa, TK/VA/67, CK/PA/9801289) have a deletion in HA1 from amino acid 230 to 238, which has been previously described as a feature of the LBM lineage [10,11].

Seven of the 12 isolates were the N2 NA subtype (Table 1) with range of nucleotide (nt) identity of 99.0 to 91.3% (additional file 1). A stalk deletion was observed in five isolates (CK/NY/3112, CK/PA/9801289, GH/MA/148081, TK/VA/67, and CK/MD/MinMa) from amino acids 58 to 73, and one isolate, TK/NY/4450, had a stalk deletion from 50-73 (Table 1). Three N3 isolates shared 95.0 to 97.7% nt identity. All N3 NA genes phylogenetically assort with other North American N3 genes (additional file 1). The viruses with NA types 8 and 9, both were found to be similar to other North American wild bird origin NA genes of the same subtype (additional file 1).

All internal protein genes were similar to those of other North American AI virus isolates, but showed some phylogenetic diversity among the isolates (additional file 2). Among the M genes of the 12 isolates there was 92.5 to 99.4% nt identity. Seven isolates had subtype B NS gene (93.5 to 99.3% nt identity) and five were subtype A (96.2 to 98.3% nt identity) (table 1). The NP genes ranged in nt identity from 91.7 to 99.2%. The PA, PB1 and PB2 genes had 86.3 to 99.0%, 91.5 to 99.1% and 88.6 to 99.2% nt identity, respectively.

## Discussion

Twelve H7 LPAI viruses were evaluated for their pathogenesis in chickens, ducks and turkeys. The general patterns of virus shed observed here; OP shed peak at 2-4 days PI and overall higher tittered OP than CL shed which peaked later, at 4-7 days PI, is typical of LPAI virus infections in chickens and turkeys following respiratory inoculation [13]. Similarly, clinical signs, microscopic lesions, and patterns of IHC staining were consistent with what has been reported previously [13]. The comparison among the species did show consistent differences among the species in disease severity and virus shed. Although there were no statistically significant trends in disease severity by virus isolate (although most of the genes in 9 of the 12 isolates were relatively closely related to each other) there was a clear and significant trend for clinical disease to be more severe in turkeys than in either chickens or ducks. In turkeys the mean maximum clinical scores, which included mortality, were significantly higher than those of either chickens or ducks with 8 of 12 isolates. Mortality in turkeys possibly had a secondary bacterial component, which is typical in the field with respiratory viruses. Importantly, mortality was not observed in the controls, varied among the AIV isolates, and the birds which died shed the highest virus titers, therefore it appears that AIV was a critical factor for turkey mortality. Additionally, the turkeys generally shed significantly more virus from both the OP and CL routes at numerous time points. This suggests that turkeys may be more susceptible to disease from LPAI virus than chickens or ducks. This is consistent with a report by Tumpey et al. where turkeys were reported to be more susceptible than chickens to LP H7N2 AIV from the 2002 Shenandoah Valley outbreak [14]. In contrast, Ladman et al. [15] reported that chickens were more susceptible to disease, when inoculated with LP H7N2 AI viruses isolated from chickens when exposed by the conjunctival sac route.

Although all of the individual birds did not seroconvert, serology indicated that each of the species did become infected at the dose administered and not all birds that seroconverted developed disease. Ducks had the lowest seroconversion rates, interestingly three of the five isolates with 100% seroconversion rates were the WB isolates. In contrast turkeys had the highest rates of seroconversion, which correlated with the trend for disease in turkeys to be more severe than in either chickens or ducks. This suggests that virus dose may have been a factor in disease development.

Also, consistent with the differences in disease severity among the species, susceptibility studies have reported the 50% bird infectious dose (BID_50_) for A/Turkey/VA/158512/2002 H7N2, which was isolated from the same outbreak as TK/VA/67, to be 10^2.8^EID_50 _in chickens, 10^0.8^EID_50 _in turkeys and 10^3.5^EID_50 _in Pekin ducks [16], indicating that it is best adapted to turkeys. In contrast the BID_50 _for ML/OH/421 has been determined to be 10^6.6^EID_50 _in chickens, and10^1.0^EID_50 _in mallard ducks (data not published). Interestingly, ML/OH/421 had some of the lowest shed titers of all the viruses in all three species and was shed at the highest titers by turkeys. Turkeys also had the highest clinical scores of all three species with ML/OH/421. Importantly, it is unknown how the chicken egg passage that was used to propagate these viruses may affect adaptation to chickens and turkeys.

These results are similar to recent comparable studies with 20 H5 subtype LPAIV viruses [17], where turkeys were more susceptible to infection and disease, however mortality was not observed with the H5 isolates. Another comparable study with 16 H4, H6 and H9 subtype viruses [18] did not show differences in disease between chickens and turkeys, but based on serology, turkeys could be infected with more viruses that chickens. In contrast, taking into account data from Halvorson et al. [19] that showed that not all duck origin AI viruses will readily transmit to turkeys, it is unclear whether turkeys are more susceptible to infection with influenza or are just more susceptible to disease when they do become infected.

The details of species susceptibility to LPAIV infection and disease needs to be explored with reverse genetics which can more definitively identify markers of host restriction and virulence. Additionally, minimum infectious dose studies would be necessary to establish host adaptation of the isolates. In the case of the wild bird isolates one may conclude that the isolates are more duck or shorebird adapted which seems to be supported by the virus shed levels by different species in this study. However, since numerous avian species are housed in close proximity in the live-bird markets, neither the exact host passage history nor the host adaptation can be inferred from the species of origin.

One practical implication of this is that infection would likely be detected more easily in turkeys, whereas sub-clinical infection may spread unnoticed until the birds are tested prior to slaughter (currently 100% of turkey and chicken flocks are tested for AI virus prior to slaughter in the US).

Since both LPAI viruses and HPAI viruses are reported to primarily cause sub-clinical disease in wild mallard ducks, the induction of mild disease by LPAI virus in Pekin ducks which are most closely related to mallard ducks but which are bred for rapid growth, is important for the commercial duck industry. It has been reported that some Asian lineage H5N1 HPAI virus isolates can cause disease in ducks, but the severity of disease depends on duck age and species [20,21]. Two-week old Pekin ducks, like what was used here, were among the most susceptible to disease with the Asian H5N1 HPAI virus [20].

To complement the clinical data and to provide a more complete characterization of the isolates, full genome sequence was produced for all 12 isolates and a basic analysis was conducted. In depth analysis of the HA and NA genes of the H7N2 isolates has already been reported [10,11]. There was no clear correlation between gene constellation, or a particular gene and any biological or pathogenic characteristic evaluated here. Further work with reverse genetics would need to be conducted to identify markers for species adaptation and virulence.

## Conclusions

Twelve LPAI viruses of the H7 subtype were evaluated in chickens, turkeys, and ducks for clinical disease and virus shed. All 12 viruses could infect all three species at the dose of 10^6^EID_50 _by the simulated respiratory route based on seroconversion. Turkeys consistently presented with the most severe disease and highest OP and CL shed titers, which indicate that, broad breasted white turkeys may be more susceptible to disease from some LPAI virus than white leghorn chickens or Pekin ducks.

## Methods

### Viruses

Twelve North American origin LPAI viruses were selected to represent different H7 HA genetic groups, different species of origin and different dates of origin within the available H7 subtype domestic LPAI viruses (Table 1). Viruses were propagated and titrated in 9 to 11 day-old embryonated chicken eggs by standard procedures [22].

### Pathogenesis studies

Pathogenesis studies were conducted with specific pathogen free (SPF) white leghorn chickens (*Gallus gallus domesticus*), broad breasted white turkeys (*Meleagris galopova*) and Pekin ducks (*Anas platyrhynchos domesticus*). Chickens were obtained at 2 weeks of age from a commercial supplier of SPF animals (Charles-River SPAFAS, Franklin, CT) and were housed in isolators until they were exposed to the virus at 4 weeks of age. There are no sources of turkeys and ducks which are maintained as SPF or free from viral or bacterial respiratory diseases therefore turkeys and ducks were obtained from commercial hatcheries at hatch and were housed in isolators until they were exposed to virus at 2 weeks of age. All birds were obtained from flocks with no antibody or prior exposure to AI virus. The birds were housed in glove-port isolators (Allentown Caging, Allentown, NJ) with *ad libitum *access to feed and water before and after exposure to the viruses. Ducks and turkeys were exposed to the virus at 2 weeks instead of 4 weeks of age to accommodate the larger size and faster growth rates of these species in the animal facilities. Since the immune systems of all three species at these ages are considered to be relatively immature, this difference is not expected to impact species associated differences in susceptibility to LPAIV infection and disease. Animals were cared for in accordance with established humane procedures and biosecurity guidelines.

Thirteen to 15 of each species were inoculated with 10^6 ^EID_50 _per bird in 0.1 ml by the intrachoanal route. The birds were monitored daily for clinical disease signs which were scored as follows: 0 = no signs, 1 = mild to moderate respiratory signs (mild depression in ducks), 2 = moderate to severe (i.e. depressed, not eating, neurological signs), 3 = Dead. Oro-pharyngeal and CL swabs were each collected at days 2, 4, 7, 10 and 14 post inoculation (PI) to evaluate virus shed by quantitative real-time RT-PCR (qr-RT-PCR). Three days PI, 3-5 birds from each group were euthanized and necropsied to evaluate gross lesions. Tissues (heart, lung, pancreas/duodemun, kidney, liver, ileum, jejunum, ceca, bursa, thymus, spleen, breast muscle, thigh muscle, brain, nasal cavity, adrenal glands, cecal tonsils, trachea, and reproductive organs) were collected for microscopic evaluation. Serum was collected from ducks at 18 days PI and from chickens and turkeys at 21 days PI to confirm infection status.

### qrRT-PCR

RNA was extracted with the MagMAX-96 Viral Isolation Kit (Ambion Inc. Austin, TX) with the KingFisher (Thermo-Fisher Scientific, Waltham, MA) magnetic particle processor in accordance with the manufacturer's instructions. Quantitative real-time RT-PCR was conducted with a primer-probe set that targeted the matrix gene as described previously [23] using the AB 7500 FAST (Applied Biosystems, Foster City, CA) instrument and the AgPathID (Ambion) one-step RT-PCR kit in accordance with kit instructions. Standard curves for virus quantification were established with RNA extracted from dilutions of the same titrated stock of the virus being evaluated.

### Immunohistochemistry

Because of the sensitivity limitations of immunohisotochemical (IHC) staining for AI virus antigen, tissues were only processed for IHC from birds with OP or CL shed titers greater than 10^4^EID_50 _2 days PI. Tissue sections were cut (4 μm thick) from paraffin-embedded tissue samples and mounted on charged glass slides (Superfrost/Plus; Fisher Scientific). Deparaffinization, antigen retrieval and blocking procedures have been previously described [24]. A 1:2,000 dilution of a mouse-derived monoclonal antibody (P13C11) specific for a type A influenza virus nucleoprotein (developed at Southeast Poultry Research Laboratory, USDA) was applied and allowed to incubate for 2 hours at 37°C. The primary antibody was then detected by the application of biotinylated goat anti-mouse IgG secondary antibody using a biotin-streptavidin detection system (Supersensitive Multilink Immunodetection System, Biogenex). Fast Red TR (Biogenex) served as the substrate chromagen, and hematoxylin was used as a counterstain.

### Serology

Antibody collected at 18 (ducks) or 21 (chickens and turkeys) days PI from surviving birds was used to confirm infection status. Sera were tested by commercial ELISA (FlockCheck, IDEXX Inc., Westbrook ME). Sera were tested at the manufacturer's recommended dilution of 1:500 and also at 1:100 and 1:50. The dilution of 1:100 was selected for final analysis because at this dilution there were no false-positives among the sera from negative control birds and there appeared to be better sensitivity.

### Sequencing and Phylogenetic analysis

Full genome sequencing of all isolates was performed as previously described [11]. Genbank accession numbers for new sequence generated for this study are and previously reported sequences are provided in Additional File 3. Phylogenetic analysis was performed using either ClustalV or ClustalW (Lasergene 7.1, DNASTAR, Madison, WI). Trees were constructed with BEAST v. 1.4.8 [25] using HKY substitution, empirical base frequency, Gamma heterogeneity, codon 2 partitions, relaxed lognormal clock, Yule Process tree prior with default operators with UPGMA starting tree and MCMC length of 10^6^.

### Statistical analysis

Mean maximum clinical scores were compared by poultry species for each isolate and virus shed was evaluated by species for each day PI and swab type (OP or CL). All comparisons were conducted with the Student's T-test and the Mann-Whitney rank-sum test if the normality test failed (Sigmaplot 11.0, Systat Inc. San Jose, CA). All statistics were evaluated with a significance threshold of p ≤ 0.05

## Abbreviations

AI: avian influenza; BID_50_: 50% bird infectious dose; PI: post inoculation; EID_50_: 50% egg infectious dose; HA: hemagglutinin; HP: high pathogenicity; IHC: immunohistochemistry; LBM: live bird market; LP: low pathogenicity; NA: Neuraminidase; qrRT-PCR: quantitative real-time reverse-transcription polymerase chain reaction; SPF: specific pathogen free; WB: wild bird.

## Competing interests

The authors declare that they have no competing interests.

## Authors' contributions

ES was involved in virus selection and experimental design and conducted sequence and phylogenetic analysis. JG was involved in virus selection and experimental design. BL and LP conducted animal experiments, collected specimens, ran qrRT-PCR, and serological assays. CP evaluated tissue sections for microscopic lesions. MJP conducted the immuno-histochemical staining and evaluation. ETM contributed to data analysis and conducted minimum infectious dose studies. All authors contributed to data analysis and manuscript preparation.

## Supplementary Material

Additional file 1 **Phylogenetic trees of the A) N2, B) N3, C) N8, D) N9 genes of viruses included in this study**. Trees were constructed with BEAST v. 1.4.8 [25] using HKY substitution, empirical base frequency, Gamma heterogeneity, codon 2 partitions, relaxed lognormal clock, Yule Process tree prior with default operators with UPGMA starting tree and MCMC length of 10^7^. Click here for file

Additional file 2 **Phylogenetic trees of the A) NS, B) M C) NP D) PA, E) PB1 and F) PB2 genes of viruses included in this study**. Trees were constructed with BEAST v. 1.4.8 as described for additional file 1. Click here for file

Additional file 3 **GenBank accession numbers for all genes for isolates evaluated in this study.** Table of GenBank accession numbers by gene and isolate.   Accession number for genes sequenced for this study are shown in boldface type. Click here for file
