# Supplementary figures and images for: The pathogenesis of low pathogenicity H7 avian influenza viruses in chickens, ducks and turkeys
Source: Virol J. 2010 Nov 19;7:331. doi: 10.1186/1743-422X-7-331 (PMC3002305; doi:10.1186/1743-422X-7-331)

A. N2 NA gene

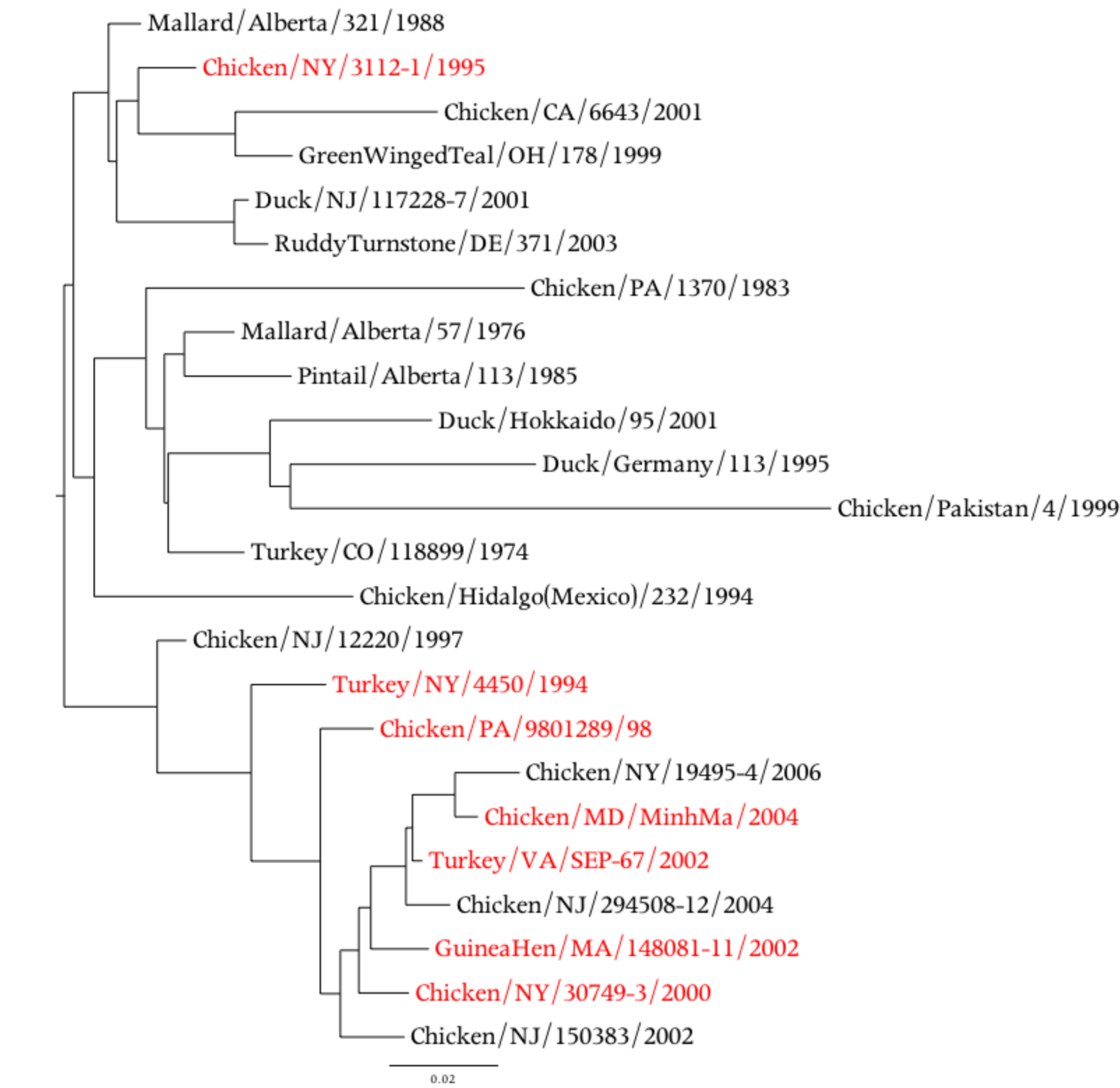

B. N3 NA gene

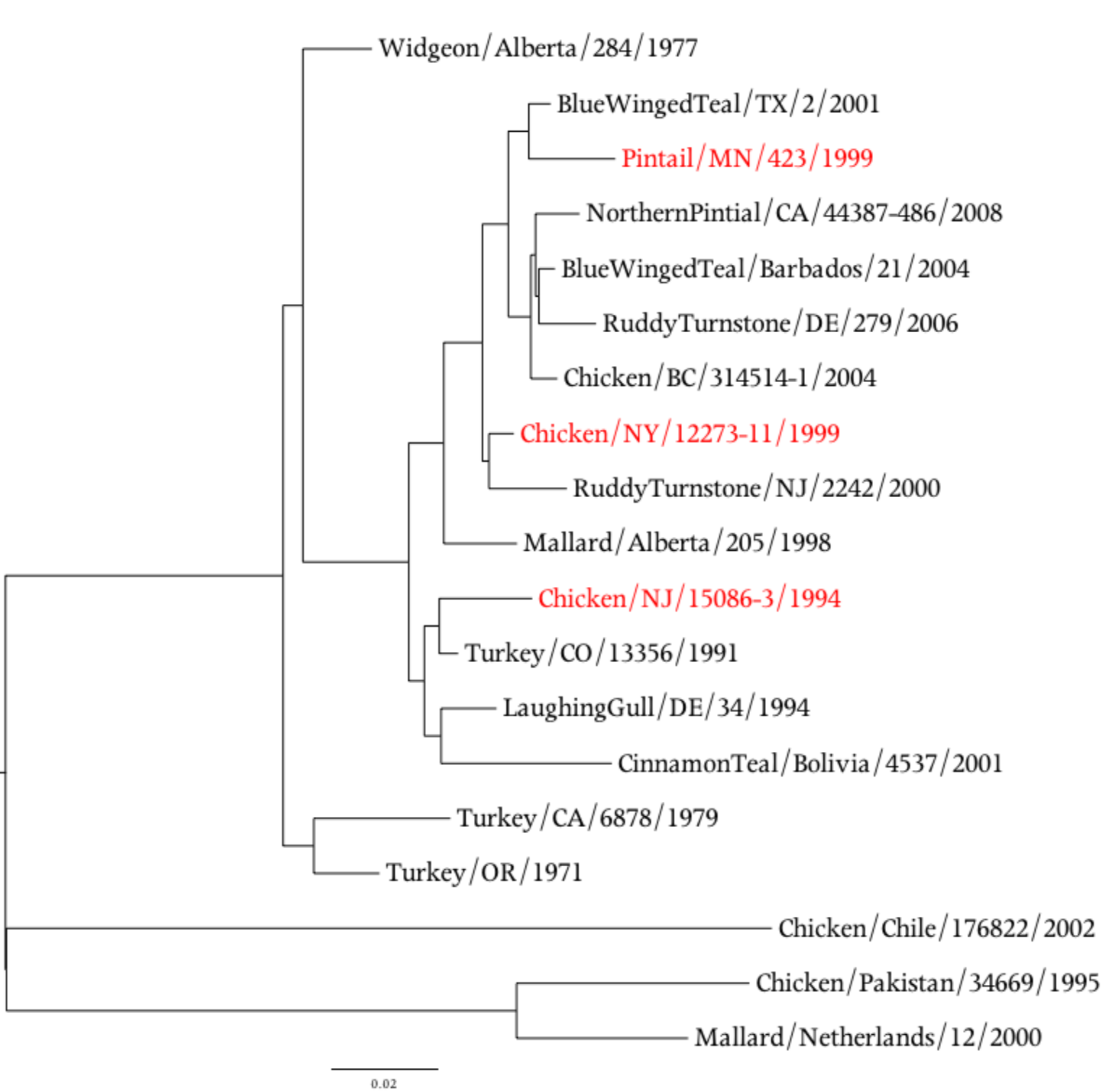

C. N8 NA gene

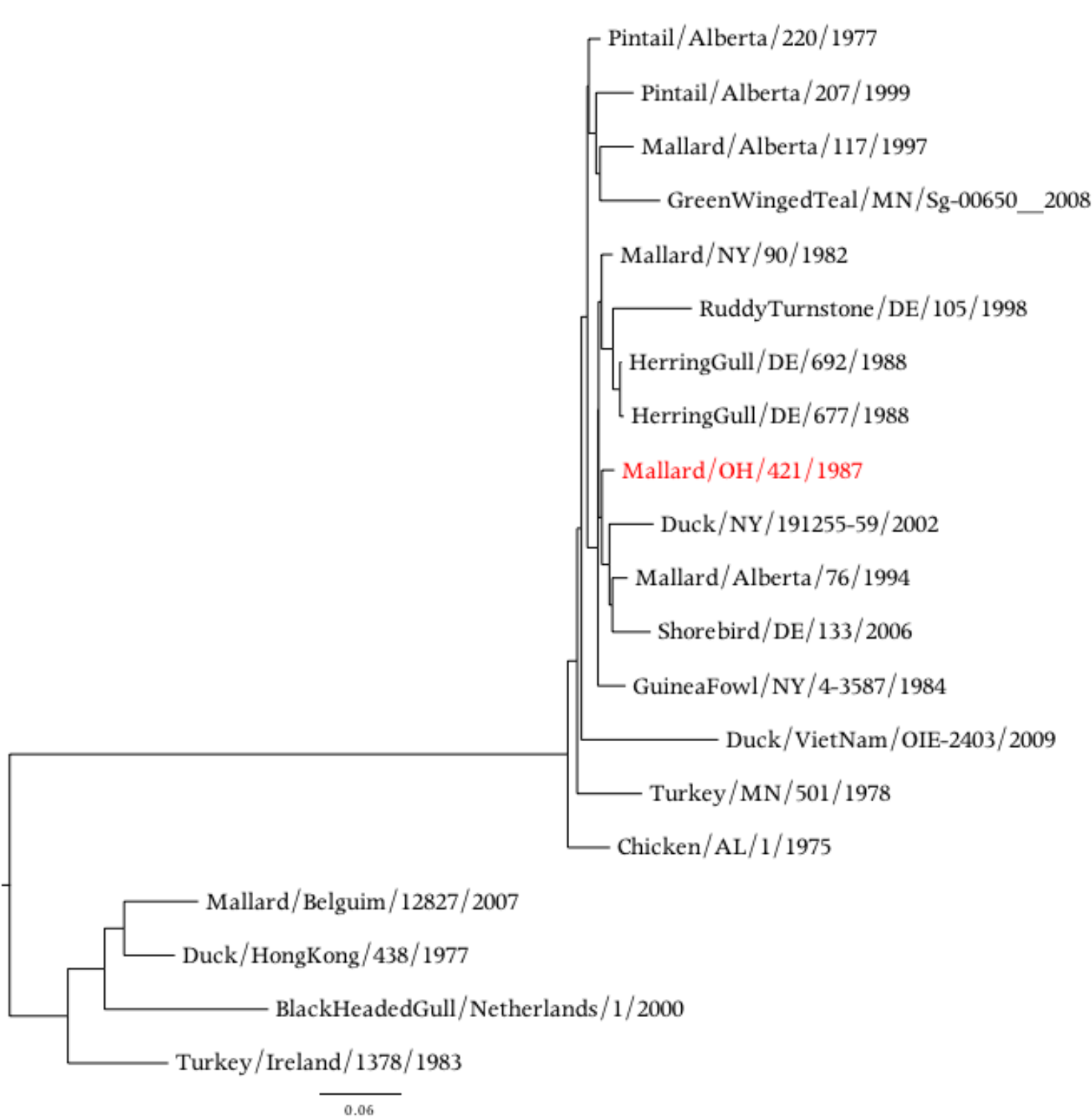

D. N9 NA gene

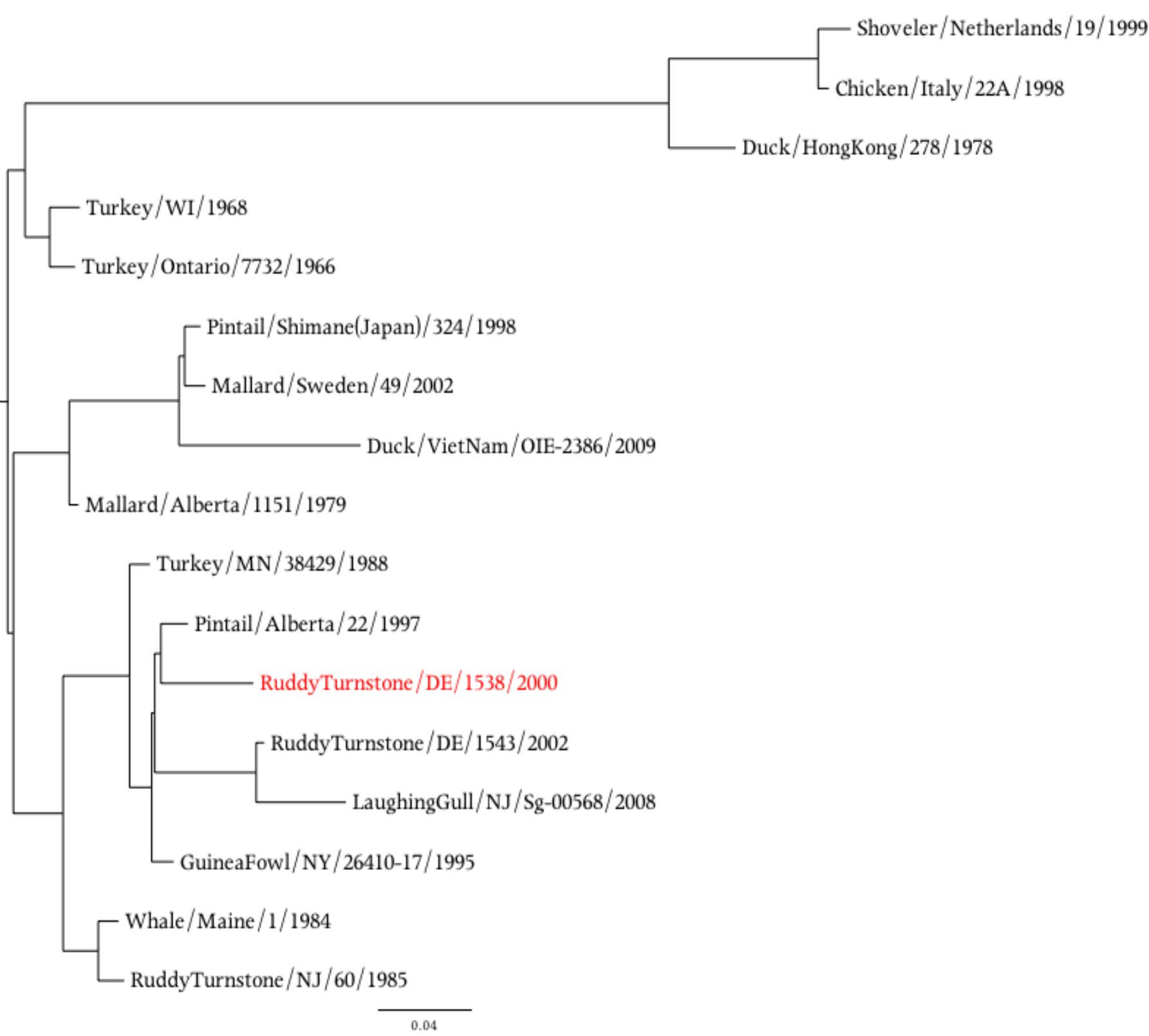

Supplement: Additional file 1 — Phylogenetic trees of the A) N2, B) N3, C) N8, D) N9 genes of viruses included in this study. Trees were constructed with BEAST v. 1.4.8 [25] using HKY substitution, empirical base frequency, Gamma heterogeneity, codon 2 partitions, relaxed lognormal clock, Yule Process tree prior with default operators with UPGMA starting tree and MCMC length of 107. [file 1743-422X-7-331-S1.PDF]

A. NS gene

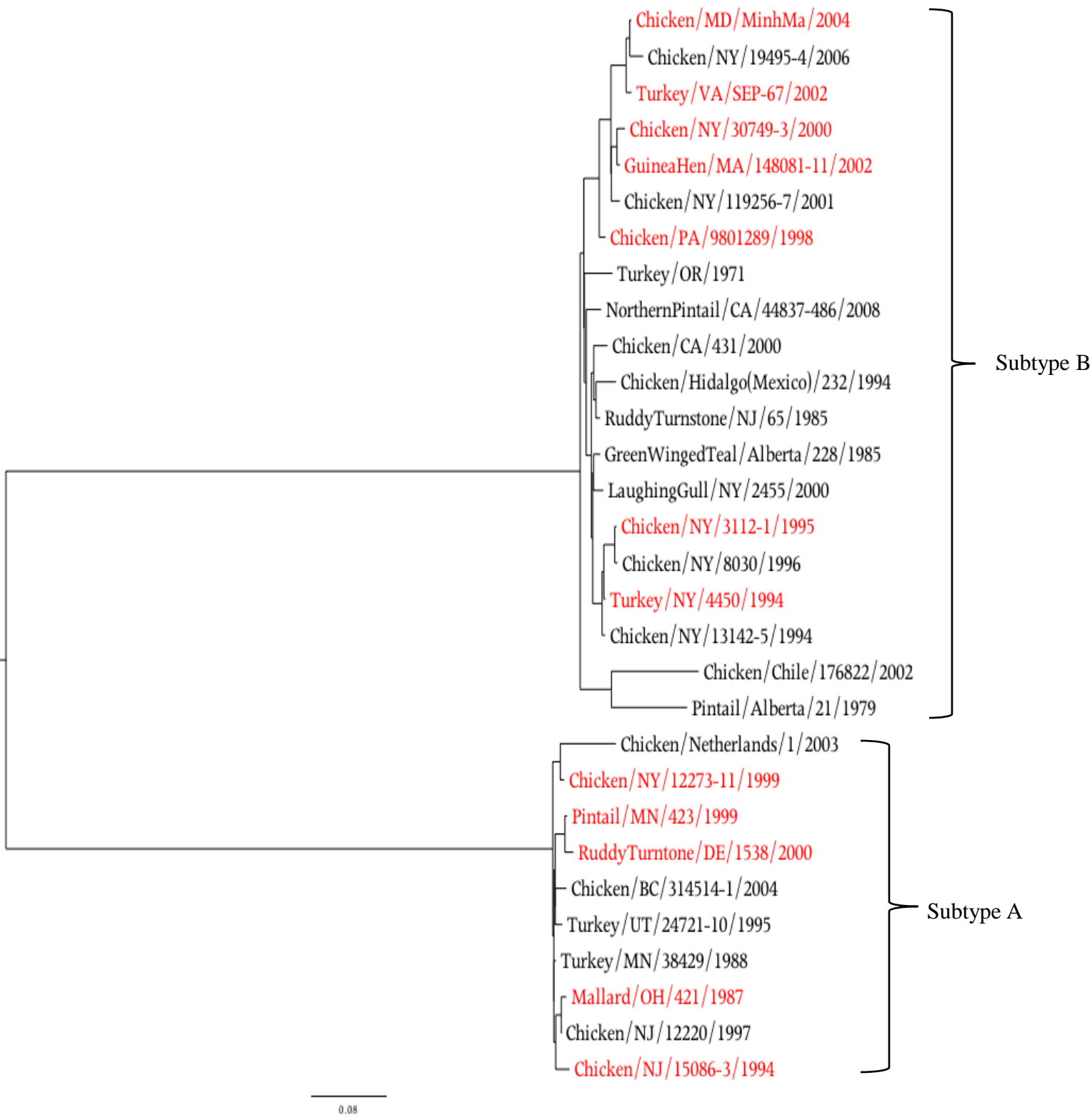

B. M gene

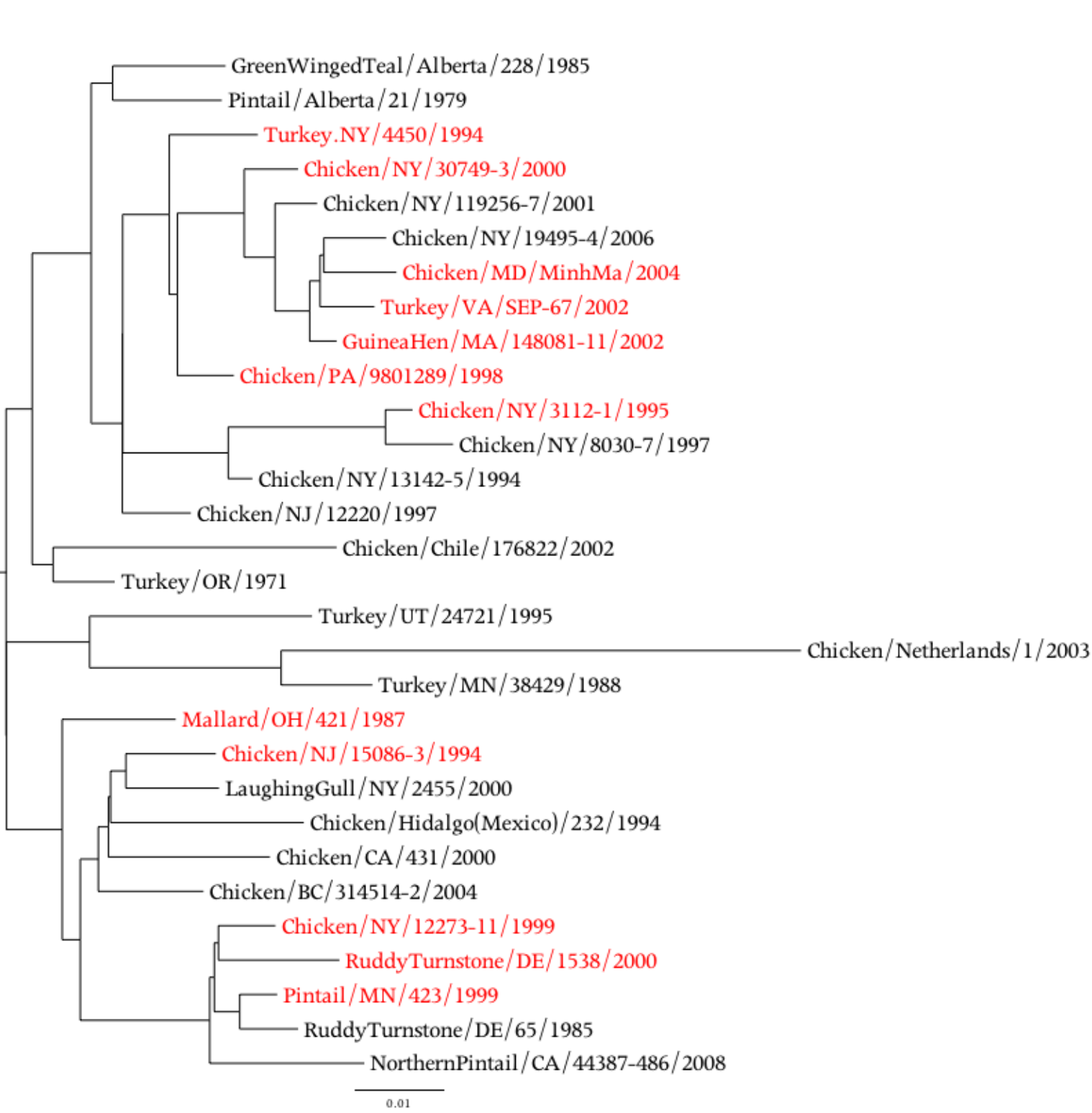

C. NP gene

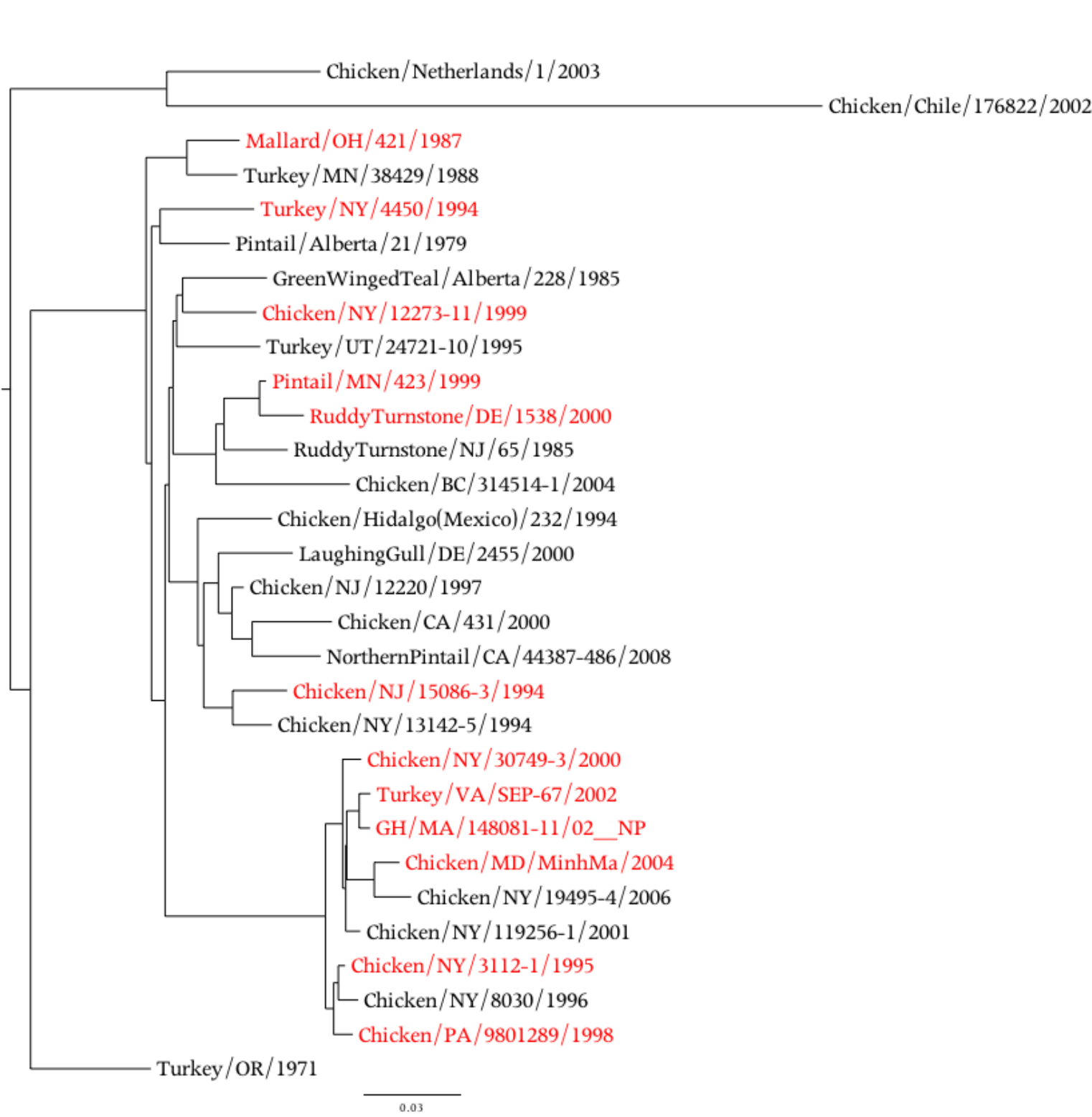

D. PA gene

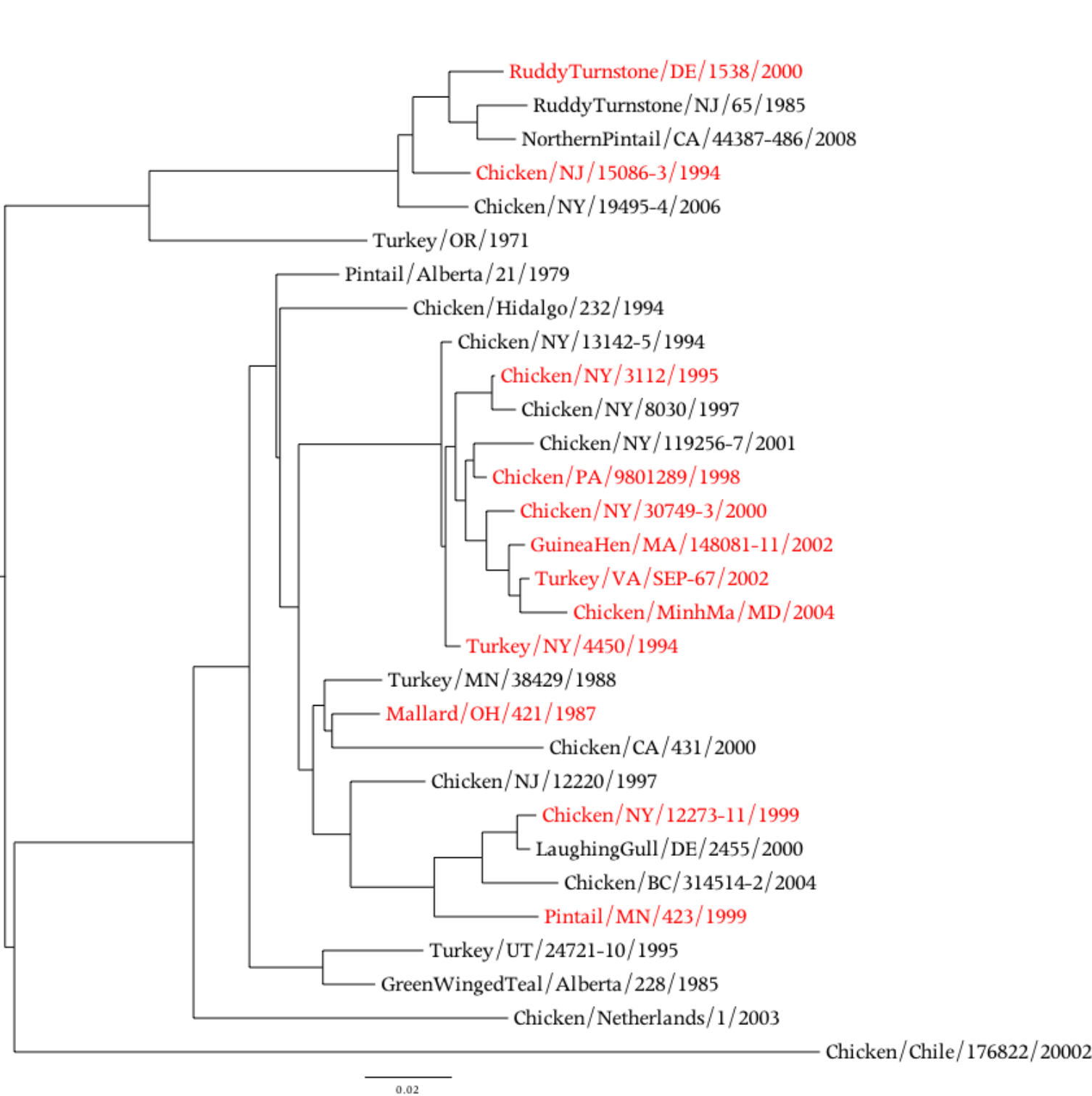

E. PB1 gene

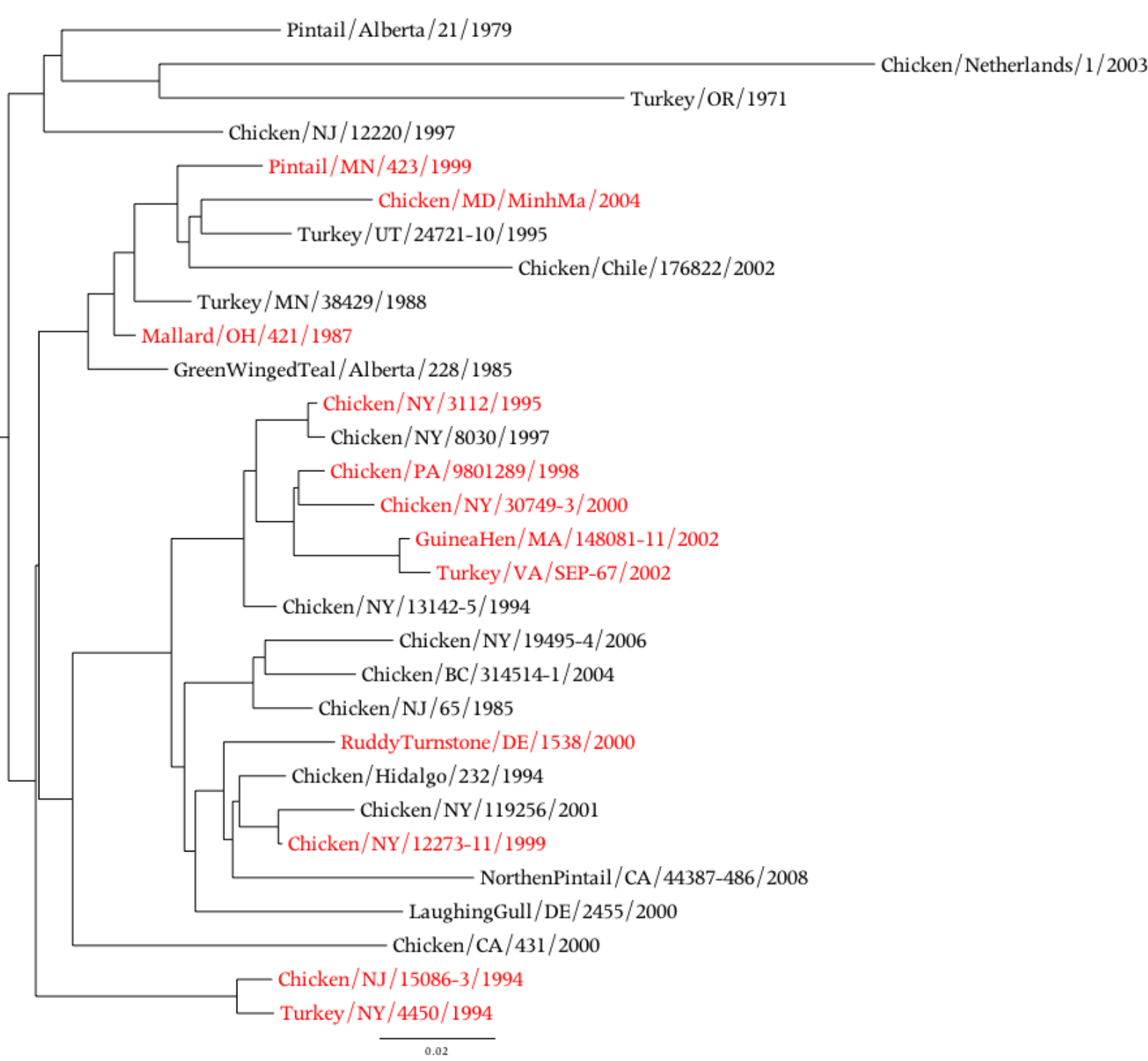

F. PB2 gene

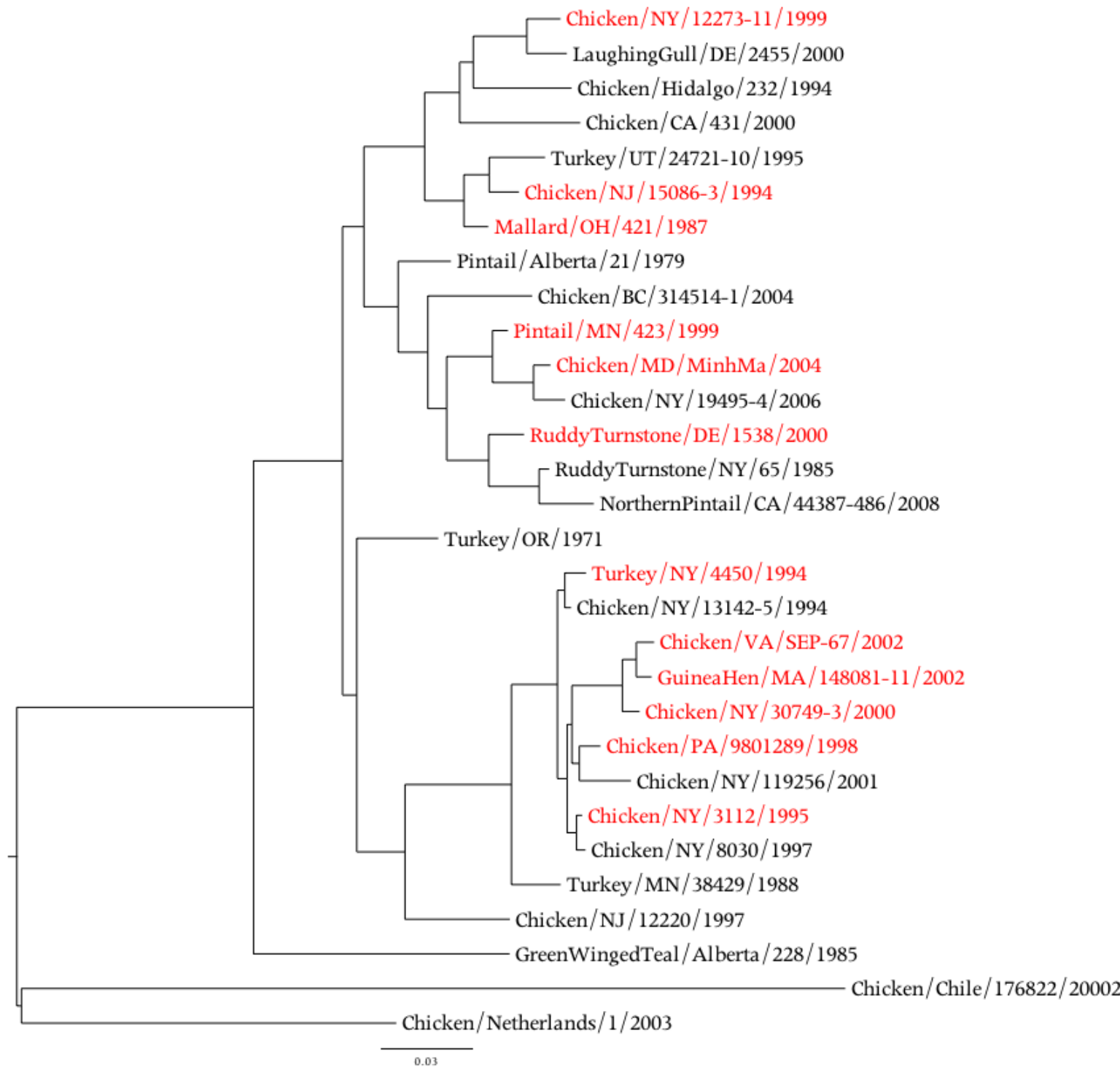

Supplement: Additional file 2 — Phylogenetic trees of the A) NS, B) M C) NP D) PA, E) PB1 and F) PB2 genes of viruses included in this study. Trees were constructed with BEAST v. 1.4.8 as described for additional file 1. [file 1743-422X-7-331-S2.PDF]
