# Supplementary material for: The pathogenesis of low pathogenicity H7 avian influenza viruses in chickens, ducks and turkeys
Source: Virol J. 2010 Nov 19;7:331. doi: 10.1186/1743-422X-7-331 (PMC3002305; doi:10.1186/1743-422X-7-331)
Supplement: Additional file 3 — GenBank accession numbers for all genes for isolates evaluated in this study. Table of GenBank accession numbers by gene and isolate. Accession number for genes sequenced for this study are shown in boldface type. [file 1743-422X-7-331-S3.PDF]

Additional file 3. GenBank accession numbers for all genes for isolates evaluated in this study. Accession number for genes sequenced for this study are shown in boldface type.

Isolate	Subtype	HA	NA	NS	M	NP	PA	PB1	PB2
A/chicken/NJ/15086-3/1994	H7N3	AF072383	AY300950	AF074267	AF073180	EU743015	EU743016	EU743017	EU743018
A/turkey/NY/4450-5/1994	H7N2	AF072386	AY254144	AF074269	AF073183	EU980461	EU980462	EU980463	EU980464
A/chicken/NY/3112-1/1995	H7N2	AF072390	AY254124	AF074273	AF073187	EU084908	EU182291	EU182290	EU084907
A/chicken/NY/12273-11/1999	H7N3	AY240892	**HQ541712**	AY241639	AY241600	**HQ541713**	**HQ541714**	**HQ541715**	**HQ541716**
A/chicken/NY/30749-3/2000	H7N2	AY240897	AY254123	AY241645	AY241606	EU084906	EU084905	EU084904	EU084903
A/guinea hen/MA/148081-11/2002	H7N2	AY240908	AY254137	AY241654	AY241617	EU742974	EU742975	EU742976	EU742977
A/chicken/PA/9801289/1998	H7N2	AY240905	AY254135	AY241652	AY241613	**HQ541717**	**HQ541718**	**HQ541719**	**HQ541720**
A/turkey/VA/SEP-67/2002	H7N2	AY240914	AY254148	AY241662	AY241624	**HQ587051**	**HQ587052**	**HQ587053**	**HQ587054**
A/chicken/MD/MinhMa/2004	H7N2	AY831670	AY831673	**HQ541722**	**HQ541721**	**HQ541723**	**HQ541724**	**HQ541725**	**HQ541726**
A/mallard/OH/421/1987	H7N8	CY021621	CY021623	CY021625	CY021622	CY021624	CY021626	CY021627	CY021628
A/pintail/MN/423/1999	H7N3	GU051495	**HQ541727**	GU051497	DQ021732	GU051496	**HQ541728**	**HQ541729**	**HQ541730**
A/ruddy turnstone/DE/1538/2000	H7N9	EU684261	**HQ541731**	DQ021611	DQ021735	**HQ541732**	**HQ541733**	**HQ541734**	**HQ541735**
